# Back to basic: Trials and tribulations of alkalizing agents in cancer

**DOI:** 10.3389/fonc.2022.981718

**Published:** 2022-11-14

**Authors:** Robert J. Gillies, Arig Ibrahim-Hashim, Bryce Ordway, Robert A. Gatenby

**Affiliations:** ^1^ Department of Cancer Physiology, H. Lee Moffitt Cancer Center, Tampa, FL, United States; ^2^ Department of Radiology, H. Lee Moffitt Cancer Center, Tampa, FL, United States; ^3^ Department of Integrated Mathematical Oncology, H. Lee Moffitt Cancer Center, Tampa, FL, United States

**Keywords:** cancer, buffer therapy, clinical trial, alkalizing, acidosis

## Abstract

“Dysregulated” metabolism is a characteristic of the cancer cell phenotype. This includes persistent use of glycolytic metabolism in normoxic environments (Warburg effect) leading to increased acid production and accumulation of protons in the interstitial space. Although often thought to be disordered, altered cancer metabolism is the outcome of intense Darwinian selection and, thus, must have evolved to maximize cancer cell fitness. In an evolutionary context, cancer-induced acidification of the microenvironment represents a niche construction strategy to promote proliferation. Ecological advantages conferred on the cancer population included remodeling of the extracellular matrix to promote local invasion, suppression of potential competitive proliferation of fibroblasts, and suppression of host immune response. Preclinical data demonstrates that increasing the serum buffering capacity (through, for example, oral sodium bicarbonate and TRIS) can neutralize the acidic tumor microenvironment with inhibition local invasion and proliferation which can be synergistic with the effects of chemotherapy and immunotherapy agents. Here, we describe the proton dynamics in cancer and their influence on tumor progression and metastasis. Additionally, we will discuss targeting the tumor acidosis with alkalizing agents including our bicarbonate clinical trial results.

**Clinical Trial Registration:**

clinicaltrials.gov, identifier NCT01350583, NCT01198821 and NCT01846429.

## Introduction

Due to a mis-match between glucose fermentation and perfusion the extracellular pH (pHe) of the tumor microenvironment is profoundly acidic (1). This acidity, as first described by the Warburg, occurs even in the presence of oxygen (2). The acid accumulated extracellularly is removed *via* different proton transporting systems, including sodium hydrogen exchanger, monocarboxylate transporters (3). This proton dynamics results in a decrease in pH and increase in an intracellular pH (pHi). The extracellular pH of a solid tumor can reach 6.5 (4). These conditions are highly toxic to normal mammalian cells and, thus, the cancer phenotype must evolve adaptive strategies to survive and proliferate in acidic conditions.

While the Warburg effect (i.e., “aerobic metabolism” - maintaining inefficient glycolytic metabolism even in the presence of oxygen) is often described as “dysregulated”, this view is inconsistent with the evolutionary model of cancer. That is, because cancer cells are subject to constant Darwinian selection, it’s metabolism must, in fact, represent an optimal phenotypic state that maximizes fitness. We have proposed that excess acid generation production represents a niche construction strategy that confers a competitive advantage on cancer cells by killing or suppressing the growth of potential normal cell competitors such as fibroblasts, causing breakdown of ECM to promote invasion, and blunting the immune response (5–7).

Functionally, cells can be classified as oxidative or fermentative in the tumor microenvironments. Oxidative cells will convert the lactic acid to pyruvate, that enter the TCA cycle and oxidized yielding ATP and CO_2_. Carbonic anhydrase 9 or 12 (CA9, CA12) on the outside o the cell will hydrate this CO_2_ producing HCO3^+^ and H^+^. In fermentative cells, Glucose will enter the cell *via* glucose transports 1 or 3 (GLUT-1, GLUT-3), and enter glycolysis after phosphorylated to Glucose 6-phosphate (G6P). The proton produced by this oxidative step is transported by sodium/hydrogen exchanger, NHE1, as well as CA9. The lactic acid produced *via* glycolysis is transported *via* monocarboxylate 1 and 4 transporters (MCT1/4) (8).

The extracellular acidity can negatively affect the normal tissue. Specifically, remodel extracellular matrix and allow tumor cells to invade and metastasize to surrounding and distal organs (9). It has been shown by us and others that acidity can also suppress immunity. *In vitro* and *in vivo* studies demonstrated that acidosis inhibits CD8 T cell function (10–12), and promote the pro -inflammatory macrophages phenotype (M2) (13).

Beyond invasion and metastases, tumor derived acid pH is also a known mediator of cancer-associated pain. In recent years, it is becoming apparent that metastasis-associated bone pain involves the reduction of peri-tumoral pH and activation of nociceptors, including acid-sensing ion channels, ASICs (14, 15). The two major nociceptor ASIC are the transient receptor potential vanilloid subtype 1, TRPV1, a.k.a. the capsaicin receptor (16, 17) and ASIC-3 (18). The expression of these transporters is decreased with bis-phosphonates, which have led some to speculate that the acid is derived from tumor-associated osteoclasts (19, 20). Osteoclasts exacerbate the tumor-derived acidity at the bone interface through their own export of protons *via* a Vacuolar type H-ATPase (21).

Although treatments to reduce intratumor acidity are often describe as “alkalizing agents,” this represents a misnomer. Typically, such agents are actually physiologic buffers which promote a pH near 7.4. Thus, they will tend to alkalinize the acidic tumor pH in the sense that the typically increase it toward the physiologic range. However, in normal tissue and physiological pH, such buffers will have no effect on the acid concentrations (22). Multiple studies have demonstrated that tumor acidity can be significantly improved with oral buffers, and this can reverse some of the consequences of acidity, including invasion to the surrounding tissue, distal organ metastasis, and modulation of immune function. For instance, we have proved that chronic treatment of animals with 200 mM sodium bicornate ad lib significantly decreased invasion and metastasis in spontaneous and experimental metastasis cancer models (23–26), and enhanced the effect of immune checkpoint inhibitors as well as adoptive T cell transfer (12). In our previously published work, we showed that Lysin has a pH dependent effect on prostate tumors metastasis. Lysin with a pH (8.0) below pka value has no effect on metastasis while Lysine with pH (10.4), a higher pka value, significantly decreased metastasis, clearly suggesting buffering is mediating the antimetastatic effect (27). In another study in transgenic prostate model (TRAMP), treating the mice before development of the tumor (~ 4 weeks after weaning) prevented the development of the interepithelial neoplasia (PIN), furthermore doubling the concentration of bicarbonate treatment after the development of the PIN lesions(~10 weeks after weaning) prevented tumor cells metastasis (28). While the mechanisms under the buffer therapy is still not completely revealed; we observe by histopathology that the buffer treated tumors are less invasive and more benign (29).

Targeting tumor acidity with buffer therapy is the most direct approach, particularly by oral sodium bicarbonate NaHCO_3_ or THAM. We have shown that mice can tolerate orally up to 200mM sodium bicarbonate with no changes to the systemic pH. Tumor volume at the time of the bicarbonate treatment influence the outcome. We observed no effect of sodium bicarbonate on large primary tumors compare to the small tumors (19, 22).

The anti-tumor effect of alkalinizing agents may be systemic and at the level of tumors. In fact, a milestone paper by our group (28) showed that the oral administration of sodium bicarbonate 100% prevented the development of prostate cancer in TRAMP mice, denoting that a daily alkalinization with either sodium bicarbonate or other buffers may well prevent cancer. Still, this probably occurs through a primary effect on the gastric pH since our stomach is not simply a digestive bug but rather an exocrine gland that produces H+ for the whole body, actively participating in the pH balance of our organism.

Recently, Helix BioPharma developed a target for tumor acidosis, L-DOS47, which can serve as an alternative to sodium bicarbonate. L-DOSE47 is a urase base extracted from Jack Bean that targets Carcinoembryonic antigen-related cell adhesion molecule 6(CECAM-6 antigen) overexpressed by several cancer types, such as lung, colon, and pancreatic (65). They urease enzyme will convert the urea present around the tumors to ammonia(2NH_4_+) and bicarbonate (1 HCO_3_ -) alkalizing the tumor microenvironment. L-DOS47 is now in phase I/II clinical trial (NCT02309892) in lung cancer (66).

Clinical trials using proton pump inhibitors remain the only evidence-based support for an anti-acidic approach against cancer that was reported; this includes a clinical study in patients with osteosarcoma (30); a case-control study in patients with metastatic breast cancer, in which an arm has been successfully treated with PPI alone (31); a pilot study in patients with GI cancers (32). Moreover, two clinical studies have been performed in domestic animals with malignant tumors, one combining standard chemotherapy with lansoprazole (33) and the other combining metronomic chemotherapy with lansoprazole and alkalinized water (34). These clinical results supported the evidence-based use of proton pump inhibitors as a new therapeutic approach, at least in combination with chemotherapy (35). However, three preclinical papers have shown that PPI alone had a potent anti-cancer effect in the absence of chemotherapy or other anti-tumor therapies in three different human tumors (36–38); suggesting that high dosage PPI should be considered in the future anti-cancer therapies. A clinical result partly supported this pre-clinical evidence in the above-quoted paper, a study performed on triple-negative breast cancer patients. In fact, at the end of the study, one arm of patients was treated with PPI alone compared to those left untreated, and the results showed a significant increase in OS in PPI-treated patients (31).

Moreover, three retrospective metanalysis have proposed PPI as an effective combined therapy with standard chemotherapy (39) and preventive treatment for breast cancer (40, 41). In a way, at least three reviews have proposed repositioning PPI for cancer therapy (42–44). Papers showed that alkalinization by oral administration of either a potent buffer (45) or alkalinized water (46), respectively, controlled the growth of a very aggressive melanoma and prevented the development of prostate cancer in TRAMP mice. Moreover, the control of melanoma growth was consistent with an increase in tumor pH and the treated mice’s urines, suggesting that a buffering approach exerted its role in inducing both tumor and systemic alkalinization.

Preclinical studies performed by us, and others suggested that oral sodium bicarbonate can be translated to clinic. Three clinical trials were conducted, phase I/IIa clinical trials to test the tolerability of oral sodium bicarbonate. We calculated the amount of sodium bicarbonate needed by comparing the amount of sodium bicarbonate that mice consumed which is around 4.2 mL per day (25). This was equivalent to 2.8 g/kg/d. By inter-species allometric scaling, human dose will be around 16.3 g/d for a 70kg human (47). In a clinical trial study for children with Sickle Cell Anemia, oral administration of 21 gm/day was safe and complication free (48). Side effects of overdose of sodium bicarbonate can include metabolic alkalosis, hypokalemia, hypernatremia, and metabolic disorders such as hypoxia, however, side effects for our dose proposed is not anticipated (49). Gastrointestinal irritability and discomfort, as well as poor taste were that main causes for the low compliance in all our three trials.

## Materials and methods

### Protocols

Clinical trial NCT01350583, NCT01198821 and NCT01846429 protocols are publicly available at clinicaltrials.gov.

## Results

Trial 1: Pilot Study. The first of these trials was conducted under IND106881 for powdered NaHCO_3_ for use in pain management. NCT01350583 was a palliative trial opened on 08/08/2010 entitled “A Pilot Study of Oral Bicarbonate as Adjuvant for Pain Reduction in Patients with Tumor Related Pain”. The rationale for this study was the prior observation that the major nociceptive (pain-sensing) receptor in cancer pain was TRPV, which has been shown to be an acid receptor (50). Target accrual was 28 patients for the 3 + 3 dose escalation study design. This trial accrued two female patients and was closed on 04/03/2012. Patient 1 completed her dose schedule of (0.5 g/kg/d) over 4 weeks. Patient 2 left voluntarily, withdrawing after 3 weeks. One grade 1-2 limb edema was reported and one each grade 1 nausea and vomitus were also reported. Both patients died 10 and 14 months after going off study.

Trial 2: GemTABS. The second trial was for pancreatic cancer (NCT01198821) patients being treated with gemcitabine under IND108551, entitled “A Phase I Study of Oral Sodium Bicarbonate in Patients with Unresectable Pancreatic Carcinoma Treated with Gemcitabine (Gem-TABS)” with a 27-month projected accrual of 35 patients. Gemcitabine has complex ionization behavior with an acid pKa of 11.65 (neutral below this value) and a base pKa of 4.47 (neutral above this value) indicating that its ionization state would not be altered between a native tumor pH of 6.5 or the bicarbonate treated pH of 7.0. Projected dose levels 1-4 of NaHCO_3_ were 0.3, 0.5, 0.7, 1.0 g/kg/d; with same patient dose escalation allowed after 2 weeks at dose if the patient experienced no treatment-related adverse events. The trial was opened 08/27/2010 and closed on 06/22/2011. A total of eight (8) patients were accrued to this trial. Treatment-related adverse events included diarrhea, limb edema and vomitus (**Table 1**). Half of the patients reported grade 1-3 fatigue, which was not ascribed as treatment related. One patient acquired a non-treatment related biliary tract infection, leading to hospitalization. The first three patients completed the level 1 dose with no grade 3 AEs. Escalation to dose level 2 was performed on two patients, who voluntarily withdrew from the trial after 11 and 26 days. As a consequence, subsequent patients were accrued at dose level 1. Overall Survival (OS) ranged from 44-718 days after initiation of trial, with a median OS of 220 days after consent (**Table 2**). A median OS of 170-177 days was reported in the gemcitabine monotherapy arms in the registration (51) and follow-up (52) trials. The difference between OS in the treated and bicarbonate groups was not significant.

**Table 1:** GemTABS adverse events.

	Grade 1	Grade 2	Grade 3	Grade 4
diarrhea	1		1	
vomiting	1	1		
edema		2		

**Table 2:** GemTABS accrual.

pt #	gender	final dose	days on Tx	OS (days)
1	f	1	126	239
2	m	1	56	197
3	m	1	63	88
4	f	2	11	319
5	m	2	26	201
6	f	1	28	320
7	f	1	82	718
8	m	1	15	44

These trials demonstrate multiple limitations of powdered NaHCO_3_. First, virtually every patient complained about the taste, which led to poor compliance. Second, there was an issue of dosing as, upon questioning, patients experiencing GI issues were likely taking too large of doses of NaHCO_3_, which is an emetic, at a single setting. The trial was designed so that doses were split between 3-4 equal doses throughout the day, which may have led to GI issues, such as vomitus and diarrhea.

Trial 3: PainCAPS. To mitigate these problems, NaHCO_3_ was re-formulated as 940 mg capsules under IND118182. NCT01846429 was designed as a phase I/II palliative trial entitled “A Phase I/II Study of Oral Bicarbonate as Adjuvant for Pain Reduction in Patients with Tumor Related Pain”. The phase I component included 3 patients per cohort (12 total) with escalating dose levels of 10, 20, 30 and 40 capsules taken throughout the day. Dose level 2 included a lead-in period of 3 weeks at dose level 1 prior to escalating to dose level 2. Each dose level was designed to last 3 weeks, after which patients were allowed to choose to leave the trial, stay on trial at the same dose, or escalate dose. The trial was opened on 09/10/2013 and terminated on 10/12/2015 with a final accrual of 9 evaluable subjects. 100% of the patients reported grade 1-2 GI disorders including vomitus. One had grade-2 limb edema, and one patient experienced grade 3 hypokalemia and was removed from study (**Table 3**). All nine evaluable patients stayed on study for the planned 3-weeks, and eight of these opted to continue at the same dose or escalate to dose level 2 following the initial lead in (**Table 4**). Patients were asked to maintain a pain diary, and tumor-related pain levels were recorded weekly on a scale of 1 (no pain) to 10 (excruciating). Across all participants, there was a significant (by Wilcoxon signed-rank test; trial NCT01846429) reduction in perceived pain from the baseline 5.25 ± 1.16 to 3.62 ± 1.88 (P = 0.010) within the first 3 weeks, and this level of significance was no different for patients who stayed on therapy for >6 weeks: 3.86 ± 1.54 (p = 0.013). On an individual patient basis, 4 of 9 patients had a reduction in pain score after 3 weeks that was greater than 1 S.D. from a baseline established over the first 3 measurements in the first 2 weeks.

**Table 3:** PainCAPS adverse events.

	Grade 1	Grade 2	Grade 3	Grade 4
hypokalemia			1	
GI/vomitus	23	9		
edema				

**Table 4:** PainCAPS accrual.

pt #	gender	final dose	days on Tx	OS (days)
1	f	1	36	NA
2	m	1	28	116
3	m	1	32	42
4	f	2	7	NA
5	f	2	15	NA
6	f	2	34	34
7	f	2	32	218
8	m	2	35	132
9	m	2	41	NA

## Discussion

We could not increase the dose of any of the oral sodium bicarbonate trials, because of taste, GI, and edema. Hence, we presume that sodium bicarbonate buffer monotherapy is not clinically feasible but there is some data to suggest the strategy of reducing intratumoral acidosis may have a favorable effect on cancer related pain. We suggest coupling the sodium carbonate therapy with other treatments to enhance the efficacy, this includes chemotherapy, and immunotherapy. As we mentioned previously, adding buffer therapy to immune blockade in mice increased response rates up to 3-fold (12).

A potential alternative strategy might reduce the requirement for supplement NaHCO_3_ therapy be following an “alkaline diet”, as discussed in (27, 53). One of the counteracts to the buffer therapy benefit is western diets since it is typically acidic. Hence, modifying the diet to include high protein content can add to the buffering benefit of the sodium bicarbonate. As published previously, this diet should contain low sulfur concentration because its oxidation will result in increased acidity and thus inhibit the net buffering effect. Essential amino acids have to be added to diet since it cannot by synthetized by the body (54).

The potential renal acid load (PRAL) is an effective way to measure the amount of acid produced by different types of food. Measuring the urine pH that correspond to each food dose level, coupled with the protein to potassium ratio (protein/K+) (55). This food buffering mechanism completely differ from the sodium bicarbonate buffering. The bicarbonate buffering creates “*compensated metabolic alkalosis”*, where kidneys secrete hydrogen ions because of the increased bold bicarbonate levels (56), which then lead to the increase of pHe in tumor microenvironment (57).

We have shown in multiple pre-clinical systems that oral buffers (*e.g.*, bicarbonate, imidazoles, Tris, lysine) explicitly increase tumor pH without changing systemic pH balance. These rarely affect growth of large primary or metastatic tumors but do inhibit small cancers thus preventing carcinogenesis or spontaneous metastases. To translate these studies into the clinic we started phase I/II clinical trials of buffer as a single agent in pain management trials (NCT01350583/01846429) and pancreatic cancer patients (NCT01198821). However, these trials failed to accrue due to poor compliance because of unpleasant taste and/or GI disturbances. However, data from one trial did show a decrease in tumor-related pain suggesting clinical efficacy may be significant if alternative treatment strategies can be devises. Thus, we suggest investigating pharmacological alternatives to achieve the same result (*i.e.*, reducing tumor acidity). As one possible solution to this issue, we have developed a point-based plan to achieve the same buffering with a combination of diet, supplements and buffers (Urbase^®^), that will be added to a trial with support from Anti-cancer Foundation (Brussels).

## Data availability statement

The datasets [NCT01350583/ 01846429] and pancreatic cancer patients [NCT01198821] for this study can be found in publicly the [clinicaltrials.gov].

## Ethics statement

The studies involving human participants were reviewed and approved by Moffitt Cancer Center under IND106881, the clinical trial NCT01350583. Under IND108551, the clinical trial NCT01198821. Under IND118182 the clinical trial NCT01846429. The patients/participants provided their written informed consent to participate in this study.

## Author contributions

All authors contributed to the article and approved the submitted version.

## Funding

This study was supported by grant R01 CA077575 and RO1 CA239219.

## Acknowledgments

The authors thank the editors for the opportunity to present this work.

## Conflict of interest

The authors declare that the research was conducted in the absence of any commercial or financial relationships that could be construed as a potential conflict of interest.

## Publisher’s note

All claims expressed in this article are solely those of the authors and do not necessarily represent those of their affiliated organizations, or those of the publisher, the editors and the reviewers. Any product that may be evaluated in this article, or claim that may be made by its manufacturer, is not guaranteed or endorsed by the publisher.
